# Case report and literature review: Conversion surgery for initially unresectable huge retroperitoneal liposarcoma after preoperative radiotherapy

**DOI:** 10.3389/fonc.2022.1096411

**Published:** 2023-01-06

**Authors:** Sarah Hsin Cheng, Yen-Shuo Huang, Hsin-Hua Lee, Heng-Hsuan Yen, Ying-Pei Jhong, Tzu-Yuan Chao

**Affiliations:** ^1^ School of Medicine, College of Medicine, Kaohsiung Medical University, Kaohsiung, Taiwan; ^2^ Department of Pathology, Kaohsiung Medical University Hospital, Kaohsiung Medical University, Kaohsiung, Taiwan; ^3^ Department of Radiation Oncology, Kaohsiung Medical University Hospital, Kaohsiung Medical University, Kaohsiung, Taiwan; ^4^ Ph.D. Program in Environmental and Occupational Medicine, Kaohsiung Medical University and National Health Research Institutes, Kaohsiung, Taiwan; ^5^ Department of Radiation Oncology, Faculty of Medicine, College of Medicine, Kaohsiung Medical University, Kaohsiung, Taiwan; ^6^ Center for Cancer Research, Kaohsiung Medical University, Kaohsiung, Taiwan; ^7^ Department of Radiation Oncology, Kaohsiung Municipal Siaogang Hospital, Kaohsiung, Taiwan

**Keywords:** conversion surgery, retroperitoneal sarcoma, liposarcoma, inoperable, neoadjuvant radiotherapy, adjuvant chemotherapy, giant tumor

## Abstract

**Background:**

Retroperitoneal liposarcoma (RPLS) is a rare malignancy that is notorious for recurrence. Surgical resection with clean margin is the current treatment of choice. However, owing to the large retroperitoneal space, RPLSs often grow to significant sizes before being diagnosed. Neoadjuvant and adjuvant therapies have potentials to improve long term treatment outcome.

**Case presentation:**

A 55-year-old Han Chinese male presented to the general surgery department with a one-year history of abdominal fullness and a one-week history of palpable right inguinal mass. At first, he was diagnosed with incarcerated inguinal hernia. However, abdominal computer tomography (CT) and biopsy confirmed his final diagnosis to be retroperitoneal well-differentiated liposarcoma, cT2bN0M0, stage IIb. The tumor, which measured 44.5cm in maximum diameter, was too large for primary surgical resection. Neoadjuvant radiotherapy with 70 Gy in 35 fractions was delivered to the tumor, which shrunk the target volume from 6300 cc to 4800 cc, as observed in the middle of the radiotherapy course. The right testicular mass also received 70Gy/35Fx. Conversion surgery was performed after radiotherapy. Unfortunately, due to residual tumor, adjuvant chemotherapy consisting of AIM (ifosfamide, Mesna, and doxorubicin) and MAID (Mesna, doxorubincin, ifosfamide, and dacarbazine) regimens were administered sequentially. Afterward, debulking surgery was conducted, plus another 18 cycles of ifosfamide monotherapy when residual tumor was still seen on CT. Since the completion of ifosfamide chemotherapy, the patient has been cancer free with no evidence of tumor recurrence for more than 26 months.

**Conclusion:**

Despite conflicting evidence in the literature, our case supports the use of high dose neoadjuvant radiotherapy and adjuvant chemotherapy in treating large, unresectable RPLSs. It also highlights the importance of using individualized, multidisciplinary approach in achieving cure for large, unresectable rare tumors.

## 1 Introduction

Sarcomas develop from the connective tissues and the majority grows in the extremities (1). Only about 15% of sarcomas develop in the retroperitoneum (1). Out of all retroperitoneal sarcomas (RPSs), retroperitoneal liposarcomas (RPLSs) are the most common, accounting for 41% of RPSs (1, 2), while only accounting for 0.07% to 0.2% of all neoplasms (2). World Health Organization classification system subdivides liposarcomas into 5 distinct subgroups, each with its distinct clinical behavior and aggressive potential (3). The most commonly seen subgroup is the well-differentiated liposarcoma (WDLPS), which tends to be slow-growing and with less metastatic potential (2, 4). The 5-year survival rate of WDLPS is about 90% (2). However, it is known for recurrence. Even after complete surgical resection, the 5-year local recurrence rate is still 50% (1, 2). Most patients who succumb to RPLS die from the effect of local recurrence, not from distant metastases (1, 2).

Currently, the only definitive treatment for RPLSs is complete surgical resections with negative margins (1, 2, 5). However, owing to the large space in the retroperitoneum, RPLSs tend to be quite large before patients begin to show symptoms and seek medical help (2, 5). According to an analysis, approximately half of the RPLSs are greater than 20cm at diagnosis (2). Such size and proximity to vital retroperitoneal structures limit the surgeon’s ability to achieve complete resection, a major predictor of survival and recurrence (1, 2, 5). Therefore, to improve complete resection rate, investigations into neoadjuvant and adjuvant therapies’ efficacy were of great importance. Specifically, conversion surgery, defined as surgical treatment with curable intention after an initially unresectable tumor has responded to preoperative treatment, would be particularly beneficial. Unfortunately, the rare nature of RPSs and their heterogeneity make high level evidence difficult to come by (2, 5). To date, the roles of neoadjuvant and adjuvant therapies for RPLS have remained controversial (6). In this report, we present a case of huge RPLS, whose greatest diameter reached 44.5cm at presentation. The patient was initially deemed unsuitable for primary surgical resection. However, with the addition of high dose neoadjuvant radiotherapy (NART) and adjuvant chemotherapy (AC), he was cured and has remained cancer-free with minimal adverse effect for more than 26 months. To the best of our knowledge, no other published case reports have described the preoperative and postoperative therapies for a large RPLS in such details.

This study was reported in agreement with principles of the CARE guidelines (7).

## 2 Case description

A 55-year-old Han Chinese male presented to a local hospital with abdominal fullness and a palpable right inguinal mass. Both symptoms had been present for a year, but only worsened in the past few weeks, prompting the patient to seek medical attention. The patient had a history of intracerebral hemorrhage secondary to ruptured venous aneurysm 25 years ago. He recovered well and his activity of daily living was totally independent.

**Figure 1:**
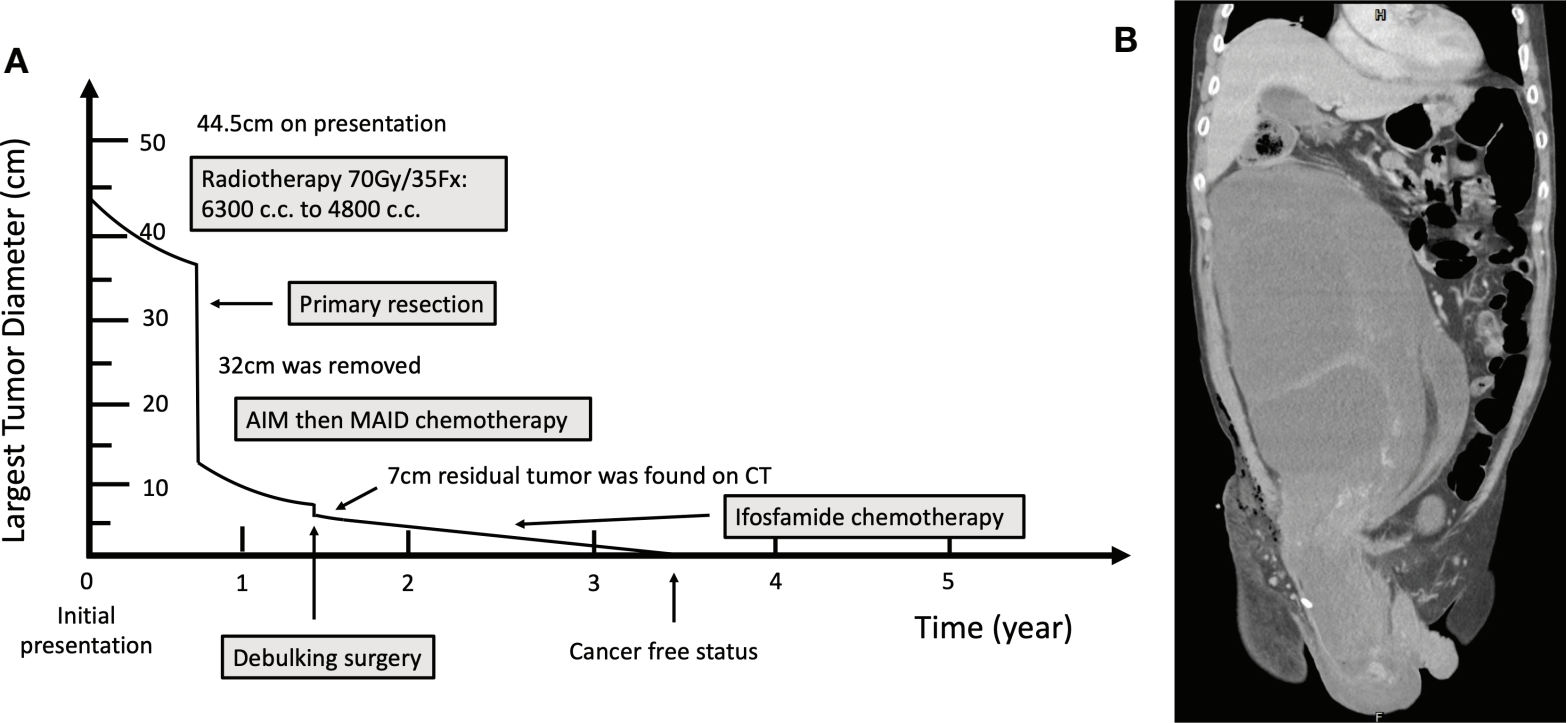
**(A)** Timeline of the occurrence of major clinical events. **(B)** Computed Tomography image before neoadjuvant radiotherapy.

**Figure 2:**
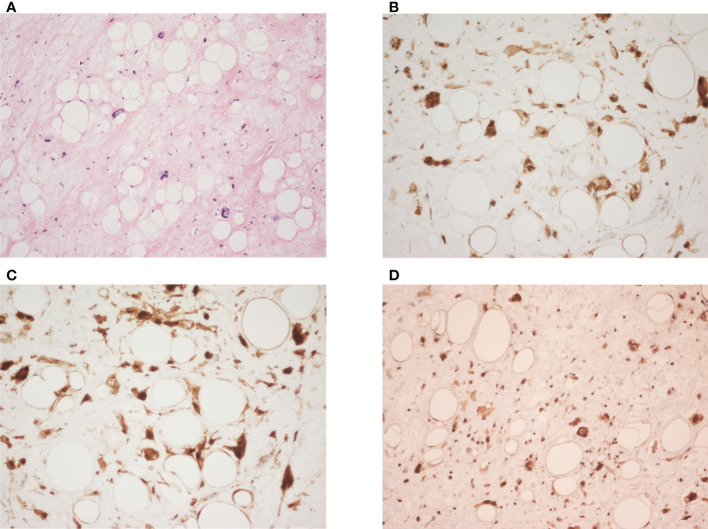
**(A)** It shows variably sized adipocytes with fibrous stroma, containing atypical cells with hyperchromatic nuclei and marked pleomorphism. (hematoxylin-eosin; original magnification ×200). **(B–D)** The neoplastic cells are immunoreactive for CDK4 (original magnification ×400), p16 (original magnification ×400), and MDM2 (original magnification ×200) respectively.

At first, the clinical impression following physical examination was incarcerated inguinal hernia. However, during the course of herniorrhaphy, bulging cord and scrotum were noted. Without prior computed tomography (CT) imaging studies, only the part of the tumor that was exposed during the surgery was excised. The excised specimen was sent for pathology studies. Subsequent abdominal and pelvic CT imaging revealed a right retroperitoneal mass with a maximum diameter of 44.5cm, protruding into the right inguinal space (**Figures 1A, B**). Histological examination of the surgical specimen showed the tumor to be composed of mature adipocytes with substantial size variation, which was appreciated alongside nuclear atypia in fat cells or stromal spindle cells. Scattered hyperchromatic and pleomorphic stromal spindle cells were easily identified within fibrous septa or blood vessel walls. Moreover, the neoplastic cells were immunoreactive for CDK4, p16 and MDM2 (**Figures 2B–D**). The final diagnosis was retroperitoneal WDLPS (FNCLCC, grade 1), cT2bN0M0, stage IIb (**Figure 2**). Following the excision of the right scrotal tumor, the patient developed abdominal fullness with vomiting and fever up to 38.5 degree Celsius. Physical examination revealed abdominal distension with tenderness and redness surrounding an oozing surgical wound. Laboratory tests revealed marked elevation in CRP and leukocytosis. Abdominal wound infection with abscess was diagnosed. Fortunately, the infection resolved after proper wound care, drainage, and antibiotic treatment.

**Figure 3:**
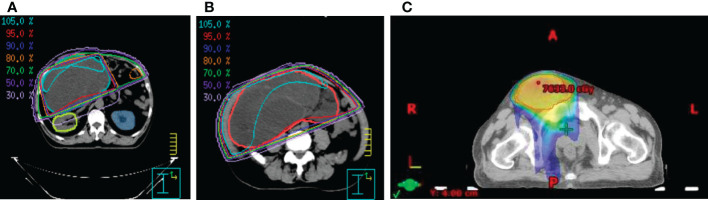
The isodose curves that show **(A)** renal parenchymal sparing and the coverage of the target tumor mass in **(B)** the peritoneal and **(C)** the inguinal area.

Due to the extensive nature of this tumor, complete surgical resection of this giant tumor was considered difficult, and primary chemotherapy was not recommended by medical oncologists. He was referred for radiotherapy at our cancer center. The organ at risk that needed to be considered first was the kidneys. Radiotherapy of 10MeV photon energy was delivered using manually chosen portals in order to maximally avoid the right kidney and completely avoid the left kidney. Thirty-five fractions of 2Gy were prescribed to the 95% isodose line. The radiation oncologist manually contoured on cross-sectional CT simulation images and measured target volume using segmentation tool programs, Eclipse and Pinnacle (**Figure 3**). The patient was monitored weekly. During the radiotherapy course, the patient reported less abdominal fullness and better digestion, which coincided with a reduction of the target volume from 6300cc to 4800cc, as noted in the adaptive treatment planning. Later on, the residual tumor mass in the right scrotum also received a total dose of 70Gy in 35 fractions (**Figure 3**). There was a grade 2 radiation-induced dermatitis in the scrotal area per the Common Terminology Criteria for Adverse Events (CTCAE) v4.0. Radiotherapy was well-tolerated without acute toxicities greater than 2. In addition, his kidney function, as measured by creatinine clearance, remained mostly the same throughout radiotherapy.

**Figure 4:**
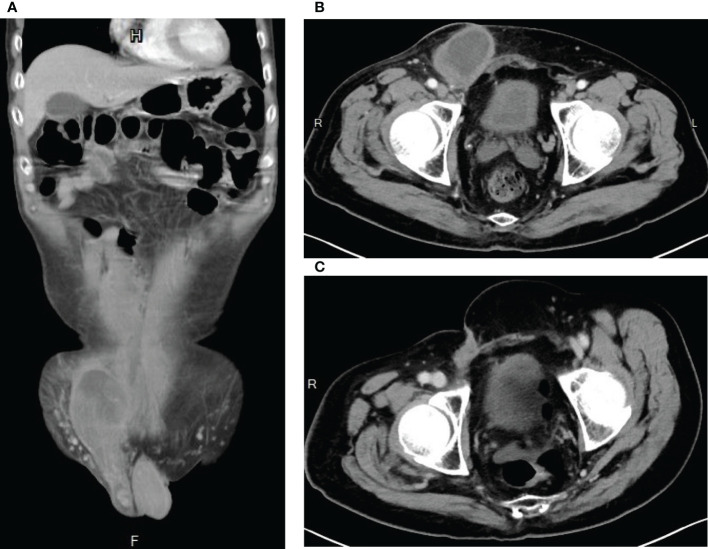
Computed Tomography images showing evidence of tumor shrinkage **(A)** after neoadjuvant radiotherapy plus conversion surgery, **(B)** after adjuvant chemotherapy AIM (ifosfamide, Mesna, and doxorubicin) and MAID (Mesna, doxorubincin, ifosfamide, and dacarbazine) regimens, and **(C)** after ifosfamide chemotherapy.

After significant volume reduction from the well-tolerated radiotherapy treatment, the patient became a candidate for surgery. He was referred to our surgical department where conversion surgery was carried out. A midline laparotomy incision was made to remove a yellowish soft, circumscribed retroperitoneal mass that measured 32cm in the greatest diameter and weighed 4.5kg. The surgery lasted around 3 hours and the blood loss was 200mL. The recovery process was smooth and no complication occurred after the surgery. However, microscopic examination revealed positive margin. Abdominal CT survey also suspected residual tumor (ycT2bN1M0, stage III) in the right inguinal area and the right pelvic wall (**Figure 4A**). Adjuvant chemotherapy was thus performed. At first, the patient received 4 cycles of AIM (ifosfamide, Mesna and doxorubicin) regimen. Then it was followed by 2 cycles of MAID regimen with Mesna, doxorubicin, ifosfamide and dacarbazine. During chemotherapy, the patient suffered from an episode of tumor site bacterial infection which was successfully treated with antibiotic and C-GSF injection. Otherwise, he mostly experienced grade 1 nausea/vomiting side effect per the CTCAE v4.0. At the completion of adjuvant chemotherapy (**Figure 4B**), the maximum tumor diameter decreased from approximately 11 cm to 8 cm. Debulking surgery for the remaining right inguinal tumor mass was thus performed. The resulting tissue fragments were 6.5cm in the greatest diameter without visible tumor cells in the section. Following the surgery, the patient developed poor wound healing, which resolved after proper wound care with Aquacel Tamponade and antibiotic use. Unfortunately, abdominal CT performed 20 days after the debulking surgery showed a 7-cm residual tumor mass in the right inguinal area and the pelvic peritoneum. This time, however, due to concern for doxorubicin’s cumulative dose-dependent cardiotoxicity (8), the patient was started on ifosfamide monotherapy only. Fortunately, after 18 cycles of ifosfamide monotherapy, no evidence of recurrence was found on abdominal CT (**Figure 4C**). He experienced infection as a chemotherapy complication early on in the ifosfamide therapy. The infection was treated promptly without major sequala. At the time of the writing of the manuscript, the patient has remained cancer free for more than 26 months.

## 3 Discussion

The role of radiotherapy in treating RPLS has been controversial. Compared to adjuvant radiotherapy, NART offers several theoretical advantages. Firstly, the gross tumor volume could be clearly defined for precise treatment planning (9). Secondly, the tumor forms a natural tissue expander, displacing radiosensitive organs, such as the small bowel, from the radiation field (2, 5, 9). As a result, dose-limiting toxicity can be minimized. Moreover, traditional principle of sarcoma radiotherapy dictates that radiation be more biologically active in the preoperative setting, allowing lower dosages to be prescribed (9). Finally, NART increases the possibility of achieving a complete resection, and thereby a cure, in tumors deemed unresectable (6). In the present case, the huge tumor was considered not suitable for either operation or chemotherapy at the discretion of surgeons and medical oncologists. Radiotherapy was his only option. It was not intended to be NART until the tumor drastically regressed and was later confirmed on adaptive treatment planning.

Indeed, these theoretical advantages seemed to have been proven in a number of small retrospective and prospective studies. NART plus curative resection were found to be better at achieving local control and prolonging survival than surgical resection only (5). Unfortunately, these studies were unable to assess the long-term benefit of NART (5, 10). To provide higher quality evidence, a randomized phase III multicenter clinical trial (STRASS) was published in 2020. Contrary to prior studies, STRASS found that NART cannot be considered a standard of care treatment for RPSs. According to the authors, abdominal recurrence-free survival (aRFS) and overall survival (OS) were similar between the NART plus surgery group and the surgery alone group at 3 years of follow-up (10). This result pointed to a lack of benefit with NART. Furthermore, STRASS also found a disproportionally high rate of adverse events among participants of the NART group, with 77% of participants experiencing grade 3-4 adverse effects (10). However, STRASS was not without criticism. Among the limitations of STRASS was defining aRFS as a composite endpoint (11). The author may have chosen such strategy so as to account for tumor progressing to unresectable during NART treatment (11). However, some critics worry that using composite endpoints would limit the ability of the study to truly capture local recurrence (11). Moreover, 3 years of follow-up was considered relatively short for a group of cancer whose 5-year local recurrence rate after complete gross resection could be as high as 80% in some histological subtypes (2). Furthermore, each histological subtype has its distinct clinical behavior. Yet, STRASS did not take such difference into account when designing the trial (11). It was not until the subgroup analysis did the authors perform a separate analysis of liposarcoma and found that in low grade sarcomas, such as WDLPS, radiotherapy seemed to have its values (10). Taken together, the benefit of NART in RPLS remains in question until higher quality evidence becomes available. However, individualized treatment incorporating NART in the present case was unexpectedly successful.

Like NART, adjuvant chemotherapy (AC) for RPLS is also poorly understood. Current evidence is contradictory with different clinical trials showing contrasting results (12). Moreover, most of the studies were done on sarcomas of the extremities (6, 12). It is still unclear the extent to which we can extrapolate the data for use in primary RPS (6, 12). An updated meta-analysis of AC on soft tissue sarcomas (STS) of any sites demonstrated marginal benefit in local, distant, and overall recurrence, as well as OS (13). Doxorubicin plus ifosfamide appeared to offer greater benefit than doxorubicin alone (13). However, subsequent large, phase III randomised controlled trial published by the European Organization for Research and Treatment of Cancer (EORTC) found no OS or recurrence benefit with ifosfamide plus doxorubicin despite the regimen being well-tolerated (14). Recent long-term study by the Italian and Spanish Sarcoma Groups shows that three cycles of adjuvant epirubicin and ifosfamide is non-inferior to five cycles of the same regimen in STS of any sites (15). As is the case with NART, more higher-level evidence is needed to understand the role of AC in treating RPLS. Fortunately, besides NART and AC, there are now some promising new treatment options on the horizon. In systemic therapy, there are molecular therapies and new synthetic agents such as marine derived synthetics, Eribulin mesylate and trabectedin (3, 16). In the field of surgical oncology, compartmental surgery is also being explored (17). In radiation oncology, off-trial results regarding STS treatments are given more attention (18). Together, they offer hope for patients battling RPLSs and its other more aggressive variants.

To the best of our knowledge, this is the first case in the literature that describes in detail the use of both high dose NART and AC to treat large WDLPS (**Table 1**).

**Table 1:** Clinicopathological characteristics of reported case of large retroperitoneal liposarcoma treated with neoadjuvant and/or adjuvant therapy in the English literature.

Authors	Publication Year	Patient Age (yr)/sex	Initial Tumor size	Tumor histology	Grade	Neoadjuvant treatment	Primary Treatment	Adjuvant Treatment	Outcome
Oh et al. (19)	2014	39/F	35 x 26 x 17cm	DDLPS	G2/3	None	Surgical resection	RT with unknown dose, then doxorubicin +Ifosfamide	No recurrence during treatment
Choi et al. (20)	2015	73/M	23 x 15 x 7cm	Myxoid	G1	None	Surgical resection	RT with 45Gy	No recurrence at 9 years
Khoury et al. (21)	2015	72/F	8.7cm	DDLPS	High grade	adriamycin/dacarbazine + 50Gy RT	Surgical resection	none	Recurred in 1 year
Kus et al. (22)	2015	40/F	20 x 15 x 10cm	DDLPS	unknown	None	Surgical resection	AIM regimen	Bone metastasis after 3 months
Zheng et al. (23)	2017	45/M	65 x 45 x 30cm	WDLPS	G1	None	Surgical resection	RT with unknown dose	No recurrence at 8 months
Da Silva et al. (24)	2018	42/F	8 x 16 x 7.7cm	WDLPS	High grade	None	Gemcitabine + docetaxel#	None	No recurrence at 8 months
Horowitz et al. (25)	2020	73/M	15 x 15 cm	DDLPS	high grade	None	Surgical resection	Doxorubicin then Palbociclib then Eribulin##	Cancer continues to progress, on palliative treatment now
Recinos et al. (26)	2021	43/M	17 cm	DDLPS	G3	None	Surgical resection	Pazopanib monotherapy*	Cancer persists with evidence of radiographic and symptomatic improvement
Nakahashi et al. (27)	2022	61/M	11cm	DDLPS	G2	None	Surgical resection	doxorubicin + ifosfamide	Recurred in 5 years
Suryabanshi et al. (28)	2022	62/M	30 x 28 x 21cm	WDLPS	Unknown	None	Surgical resection	AIM regimen	Local recurrence in 2 years**
Cheng et al., present case	2022	55/M	44.5cm	WDLPS	G1	RT with 70Gy/35fr	Surgical resection	AIM followed by MAID; then ifsofamide monotherapy	Cancer free for 26 months

#During the initial presentation, the cancer was surgically excised. However, on recurrence, chemotherapy alone was utilized to treat the recurrent tumor.

##Palbociclib then Eribulin are palliative for the patient with the goal of halting progression of the cancer.

*The patient did not tolerate postoperative radiotherapy of unknown dose and developed intolerable neurological side effect from doxorubicin and ifosfomide.

**The patient was inconsistent in follow up.

DDLPS, dedifferentiated liposarcoma; WDLPS, well-differentiated liposarcoma; RT, radiotherapy; AIM, Doxorubincin(Adriamycin), ifosfamide, and mesna; MAID, ifosfamide, doxorubicin, mesna, dacarbazine.

Due to the huge tumor size at presentation, the tumor was deemed unresectable. Without successful downstaging with a preoperative therapy, the patient had little chance of a cure. NART was thus administered, but with carefully chosen portals to prevent irradiation to critical organs. However, after surgical excision, remanent tumor mass was found on CT which could not be completed excised without causing significant morbidity to the patient. Likewise, radiotherapy would induce significant toxicity to adjacent retroperitoneal structures. Given the patient’s relatively young age and fitness, chemotherapy was deemed an appropriate option. Therefore, AC regimen containing ifosfamide and doxorubicin was administered.

Another unique point about our case is the high radiation dosage (70Gy in 35 fractions) that was used. In our review of the literature, no other published studies have used such high dosage. For comparison, STRASS trial only used a radiation dosage of 50.4Gy in either 3D-Confromal RT or intensity modulated radiation therapy (10). The dosage we used correlates to a 2006 study that suggests a theoretical dose escalation of up to 80Gy could be considered in RPLS (29). Our patient responded well to 70Gy with good adherence. Grade 2 radiation-induced dermatitis was observed during radiotherapy, which subsided soon. To date, he has not developed any chronic radiotherapy-related adverse effect since completing the treatment more than 5 years ago. Furthermore, despite WDLPS’ high recurrence rate, his cancer has not recurred for more than 2 years. The high NART dose may have contributed to his prolonged recurrence free survival since a number of studies have pointed to dose escalation as a possible solution to poor local control (29). However, the limitation of our present case report is the relatively short follow-up duration prior to its publication. As a malignancy known for recurrence, the patient has been cancer free for more than 2 years and will be followed up throughout his lifetime.

In conclusion, we present a case of RPLS who was cured by NART of 70Gy followed by surgical resection and AC. The case highlights the importance of individualized, multidisciplinary approach in treating large, rare malignancy. Although the role of NART and AC in RPLS treatment are still being debated, clinicians should not rule them out as viable options. For patients who are not candidates for standard therapies, these options, when carefully tailored, can be their only chance of a cure.

## 4 Patient perspective

I was shocked to see such a huge tumor residing inside my body on the CT scan. To put it simply, I had no debilitating symptoms, except some abdominal fullness and a visible inguinal mass. After the initial shock, the reality of treating the huge cancer sank in when surgeons and oncologists told me the tumor was too big for surgery or chemotherapy. My heart sank. By the time I had arrived at the Department of Radiation Oncology, I had lost most of my hope. However, during the course of the radiotherapy, when my bulging stomach began to flatten and I gradually regained my appetite, I began to feel hopeful again. After radiotherapy, I received an operation followed by chemotherapy. And then there was yet another surgery to the inguinal area followed by even more chemotherapy. The arduous journey took over 3 years. Along the way, there were multiple hospital visits for various treatments and checkups. Sometimes I had complications from these treatments, which further increase my hospital stays. Despite all these challenges, I survived the cancer and was declared cancer free. I am forever grateful to my family for their untiring support and to all the healthcare professionals for never giving up on me and whose expertise has allowed me a second chance at life. I feel more alive each day.

## Data availability statement

The original contributions presented in the study are included in the article/supplementary material. Further inquiries can be directed to the corresponding author.

## Ethics statement

Ethical review and approval was not required for the study on human participants in accordance with the local legislation and institutional requirements. The patients/participants provided their written informed consent to participate in this study.

## Author contributions

SC and Y-SH completed the first draft and generated graphs. H-HL designed the radiation field and critically revised the manuscript. H-HL, H-HY, Y-PJ and T-YC treated the patient and participated in treatment planning, dose calculation, and quality assurance. All authors contributed to the article and approved the submitted version.
